# Return to Work after Percutaneous Coronary Intervention: The Predictive Value of Self-Reported Health Compared to Clinical Measures

**DOI:** 10.1371/journal.pone.0049268

**Published:** 2012-11-16

**Authors:** Karin Biering, Torsten Toftegaard Nielsen, Kurt Rasmussen, Troels Niemann, Niels Henrik Hjollund

**Affiliations:** 1 Department of Occupational Medicine, Regional Hospital West Jutland, Herning, Denmark; 2 Department of Cardiology, Aarhus University Hospital, Skejby, Aarhus, Denmark; 3 Department of Cardiology, Herning Regional Hospital, Herning, Denmark; 4 Department of Clinical Epidemiology, Aarhus University Hospital, Aarhus, Denmark; S.G.Battista Hospital, Italy

## Abstract

**Aims:**

Coronary heart disease is prevalent in the working-age population. Traditional outcome measures like mortality and readmission are of importance to evaluate the prognosis but are hardly sufficient. Ability to work is an additional outcome of clinical and societal significance. We describe trends and predictors of Return To Work (RTW) after PCI and describe a possible benefit using patient-reported measures in risk stratification of RTW.

**Methods:**

A total of 1585 patients aged less than 67 years treated with PCI in 2006–2008 at the Aarhus University Hospital were enrolled. Clinical information was provided through the West Denmark Heart Registry, and 4 weeks after PCI we mailed a questionnaire regarding self-rated health (response rate 83.5%). RTW was defined at weekly basis using extensive register data on transfer payments. Predictors of RTW were analysed as time to event. ROC curves constructed by logistic regression of predicting variables were evaluated by the c-statistic.

**Results:**

Four weeks before PCI 50% of the patients were working; the corresponding figures were 25% after 4 weeks, 36% after 12 weeks, and 43% after one year. The patients’ self-rated health one month after the procedure was a significant better predictor of RTW compared to other variables including LVEF, both at short (12 weeks) and long (one year) term.

**Conclusions:**

The patient's self-rated health four weeks after the procedure was a stronger predictor than left ventricular ejection fraction (LVEF), and consequently useful when patients seek medical advice with respect to RWT.

## Introduction

In Denmark, approximately 9000 patients are treated annually with percutaneous coronary intervention (PCI), of whom 2500 are below 65 years [1]. Traditional outcome measures like mortality and readmission are of importance to evaluate the prognosis, but are hardly sufficient. Ability to work is an additional outcome of clinical and societal significance. Literature about PCI and return to work (RTW) in general is sparse and often described in selected populations with potentially inadequate measures of RTW. In Denmark, it is possible to describe RTW week by week for all citizens due to full coverage of population-based registers. A recent register-based study on labour market participation among patients with coronary heart disease showed that they left the workforce faster compared to persons without coronary heart disease [2].

LVEF is a well-described predictor of RTW [3], [4], while patient-reported health measures are less often used. Use of patient-reported measures in clinical practice has become increasingly more frequent during the last decade [5]. There is agreement that patient-reported measures add valuable information to the clinicians, but some measures are considered time-consuming and difficult to handle in the clinical setting [6]–[9]. Self-reported health and quality of life has previously been found to predict readmissions and mortality [8], [10]–[12], and it may be useful for prediction of difficulties in RTW and a useful tool in risk stratification in rehabilitation if kept simple and easy to handle.

The primary aim of the study was to describe frequency and time trends of RTW for the PCI-treated patients in an unselected working age population, and to identify predictors of RTW. Secondly, we aimed to describe the value of using simple and easily obtained patient-reported measures in individual risk stratification for RTW.

**Table 1:** Baseline characteristics of 1585 patients treated with PCI at Aarhus University Hospital 2006–2008.

		Total	Gender		P-value*	Indication		P-value*
			Men	Women		Acute	Other	
		n = 1585	n = 1256	n = 329		n = 502	n = 1083	
Age (years)	−44	8.5%	7.5%	12.2%	0.03	12.5%	6.6%	<0.01
	45–54	27.5%	28.3%	24.3%		29.1%	26.8%	
	55–59	23.4%	23.9%	21.6%		22.7%	23.7%	
	60–67	40.6%	40.3%	41.9%		35.7%	42.9%	
Gender	Female	20.8%	–	–		19.1%	21.5%	0.28
	Male	79.2%	–	–		80.9%	78.5%	
Working Status (week before PCI)	Self-supporting	37.4%	41.3%	22.5%	<0.01	52.4%	30.5%	<0.01
	Labour-market-related benefits	2.5%	2.1%	4.0%		4.4%	1.6%	
	Health-related benefits	22.8%	22.3%	24.9%		10.4%	28.6%	
	Early retirement	26.1%	23.7%	35.3%		23.5%	27.3%	
	Normal retirement	11.0%	10.4%	13.4%		9.1%	11.9%	
	Not living in DK	0.13%	0.16%	0%		0.2%	0.09%	
Indication	Acute MI	31.7%	32.3%	29.2%	0.28	–	–	
	Other	68.3%	67.7%	70.8%		–	–	
LVEF (%)	−34	5.1%	4.9%	5.8%	0.03	6.2%	4.6%	<0.01
	35–54	35.6%	37.3%	29.2%		51.0%	28.4%	
	55+	52.2%	51.0%	56.8%		33.3%	61.0%	
	Missing	7.0%	6.8%	8.2%		9.6%	5.9%	
BMI (kg/m^2^)	−24.9	28.2%	24.0%	44.1%	<0.01	30.2%	27.1%	<0.01
	25–29.9	44.0%	48.0%	28.9%		44.2%	44.0%	
	30+	23.4%	23.2%	24.0%		17.7%	26.0%	
	Missing	4.4%	4.7%	3.0%		7.5%	2.9%	
Smoking status	Never	17.6%	17.9%	16.4%	0.15	13.1%	19.7%	<0.01
	Current	42.8%	41.6%	47.1%		59.4%	35.1%	
	Previous	34.7%	35.7%	31.0%		23.3%	40.0%	
	Missing	4.9%	4.7%	5.5%		4.2%	5.3%	
Diabetes	No	83.7%	83.9%	82.7%	0.69	87.1%	82.1%	0.04
	Insuline treated	4.9%	4.7%	5.7%		6.4%	9.0%	
	Non-insuline	8.1%	8.0%	8.5%		3.6%	5.5%	
	Missing	3.3%	3.3%	3.0%		3.0%	3.4%	
Respondent to questionnaire	Yes	83.5%	83.8%	82.4%	0.55	84.1%	83.2%	0.67
	No	16.5%	16.2%	17.6%		15.9%	16.8%	

Total and stratified by gender and indication.

* P-values were derived from Chi^2^ test.

## Methods

Central Denmark Region is one of five administrative units in Denmark with 700.000 inhabitants between 25 and 67 years [13]. All patients in this region referred to acute as well as subacute PCI are treated at one single unit at the Aarhus University Hospital, Skejby. From February 2006 to March 2008, we enrolled all first-time PCI-treated patients below 67 years of age. In this period, 3966 persons were treated with PCI. Patients with no record of previous PCI (n = 1752) were identified in the hospital’s patient administrative system. Information on address and vital status was collected from the Danish Civil Registration System (CPR). A total of 167 patients were excluded because they died within the first 4 weeks (n = 26) or because CPR had recorded a denial to participate in research in general (n = 141). One month after the PCI, the remaining 1585 patients were mailed a questionnaire including questions on self-rated health (SF-12). The SF-12 instrument has previously been validated in different cohorts of heart patients [14]–[16].

**Table 2:** Patient reported outcomes 4 weeks after PCI of 1323 patients, who responded to questionnaire.

		Total	Gender		P-value*	Indication		P-value*
			Men	Women		Acute	Other	
		n = 1323	n = 1052	n = 271		n = 422	n = 901	
SF12 General Health	Poor	2.6%	2.7%	2.2%	<0.01	2.4%	2.7%	0.11
	Fair	16.3%	13.7%	26.6%		12.6%	18.1%	
	Good	40.4%	39.1%	45.8%		41.5%	40.0%	
	Very good	31.7%	35.1%	18.8%		34.8%	30.3%	
	Excellent	6.7%	7.4%	3.7%		6.6%	6.7%	
	Missing	2.3%	2.1%	3.0%		2.1%	2.3%	
SF12 Mental component score**	Mean(sd)	49.1(10.6)	50.1(10.1)	44.9(11.8)	<0.01	48.7(10.2)	49.3(10.8)	0.37
SF12 Physical component score**	Mean(sd)	45.9(10.2)	46.6(10.1)	42.6(10.1)	<0.01	46.1(9.7)	45.7(10.4)	0.55

Total and stratified by gender and indication.

* P-values were derived from Chi^2^ test or unpaired t-test.

** The SF12 component scores range from 0 to 100, the higher score the better rating of health. 156 patients had missing component scores due to one ore more missing items.

Clinical data were collected from the Western Denmark Heart Registry (WDHR), which includes data on patients who have been subjected to coronal angiography, PCI, cardiac valve and bypass operations [17]. Variables include indication of the PCI, left ventricular ejection fraction (LVEF) and body mass index (BMI).

**Figure 1:**
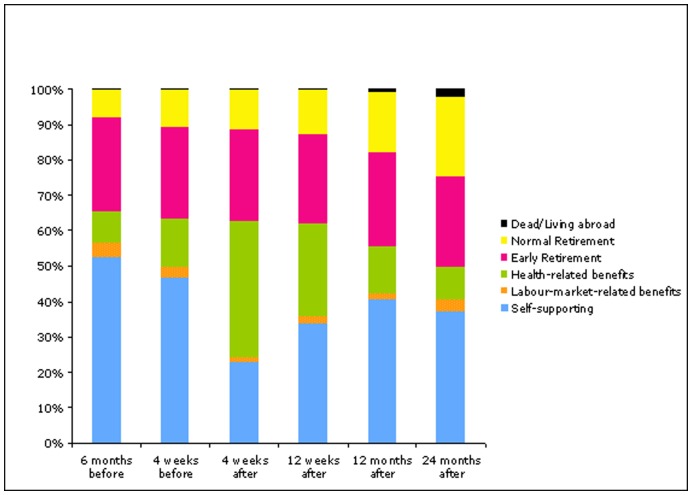
Income sources for patients before and after PCI at Skejby University Hospital 1. February 2006 to 1. March 2008.

Information on weekly working status was collected from The Danish Register for Evaluation of Marginalization (DREAM), which includes information on all public transfer payments administered by Danish ministries and municipalities for Danish citizens since 1991. In Denmark, employed citizens are entitled to sick-leave benefit after 2 weeks, and if an employer pays full salary during sick leave they receive municipal reimbursement. If there is no transfer income registered for a specific week, the person is considered self-supporting or on short-term sick leave. Data from the DREAM database has been compared to other sources of information and found valid [18].

**Figure 2:**
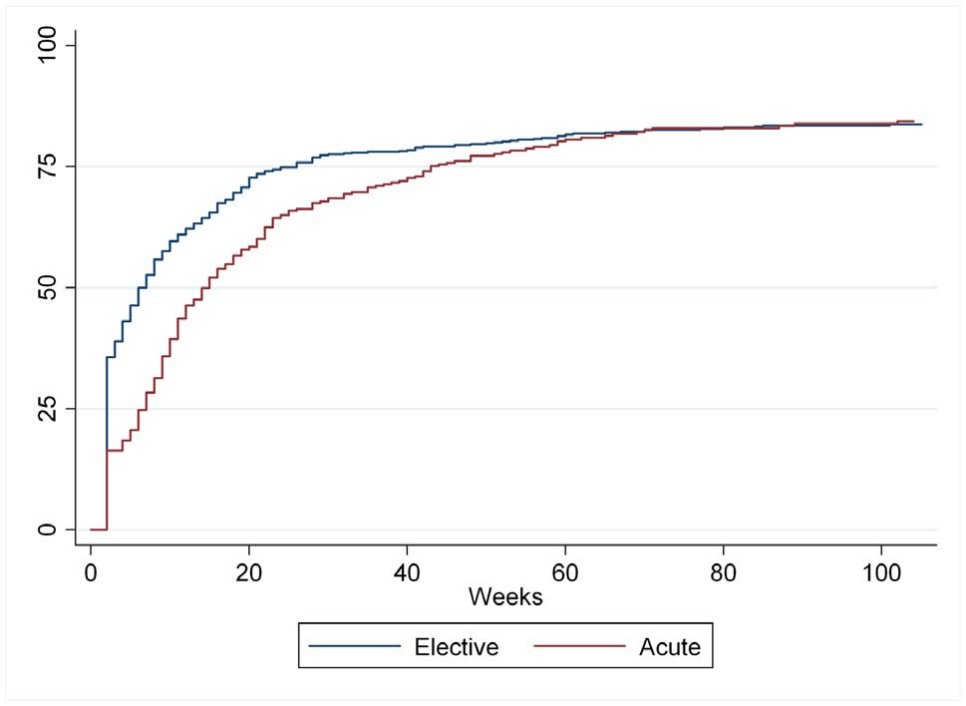
Kaplan-Meier: Return to work by indication of PCI (N = 994).

DREAM codes were grouped in 6 categories: self-supporting, labour-market–related benefits, health-related benefits, early retirement, normal retirement, and dead/emigrated. Self-supporting includes persons who receive state education fund grants, maternity leave pay, and different leave-of-absence schemes. Health-related benefits includes persons receiving social assistance with other problems than unemployment, sickness benefit, vocational rehabilitation benefit, in flex job (jobs created for persons with limited work capacity), or unemployed from flex job. Early retirement contains persons over 60 years who have retired voluntarily or who are on anticipatory pension scheme or in light job (jobs created for persons on anticipatory pension). These definitions were previously used in a validation study [18].

**Table 3:** Risk Ratio (RR) of Return to Work 12 weeks and one year after PCI according to demographics, clinical information and self-reported health.

		12 weeks after PCI		One year after PCI	
		Crude RR	Adjusted RR*	Crude RR	Adjusted RR*
Age	−44	0.69[0.54;0.87]	0.86[0.68;1.08]	0.94[0.83;1.06]	1.03[0.92;1.15]
	45–54	0.94[0.82;1.08]	1.01[0.89;1.15]	0.97[0.89;1.05]	1.03[0.95;1.10]
	55–59	0.94[0.81;1.09]	1.00[0.87;1.14]	1.00[0.92;1.09]	1.05[0.98∶1.14]
	60–67	Ref.	Ref.	Ref.	Ref.
Sex	Female	0.66[0.54;0.80]	0.72[0.58;0.90]	0.77[0.68;0.87]	0.85[0.75;0.96]
	Male	Ref.	Ref.	Ref.	Ref.
Indication	Acute MI	0.75[0.65;0.85]	0.78[0.68;0.89]	0.97[0.91;1.04]	1.00[0.94;1.07]
	Other	Ref.	Ref.	Ref.	Ref.
LVEF	−34	0.50[0.32;0.78]	0.66[0.40;1.09]	0.64[0.48;0.84]	0.71[0.53;0.95]
	35–54	0.80[0.71;0.90]	0.89[0.80;0.99]	0.90[0.84;0.88]	0.90[0.85;0.96]
	55+	Ref.	Ref.	Ref.	Ref.
SF12 GH	Poor	0.12[0.03;050]	0.14[0.04;0.58]	0.34[0.18;0.62]	0.38[0.21;0.70]
	Fair	0.45[0.34;0.60]	0.50[0.37;0.66]	0.50[0.43;0.63]	0.55[0.44;0.67]
	Good	0.66[0.56;0.77]	0.71[0.60;1.62]	0.82[0.77;0.88]	0.84[0.79;0.90]
	Very good	0.92[0.80;1.06]	0.93[0.80;1.08]	0.96[0.91;1.01]	0.95[0.90;1.00]
	Excellent	Ref.	Ref.	Ref.	Ref.
SF12 MCS	−49	0.63[0.56;0.72]	0.68[0.60;0.78]	0.79[0.74;0.86]	0.82[0.76;0.88]
	50+	Ref.	Ref.	Ref.	Ref.
SF12 PCS	−49	0.58[0.52;0.66]	0.62[0.55;0.70]	0.73[0.68;0.78]	0.76[0.71;0.82]
	50+	Ref.	Ref.	Ref.	Ref.

Low RR indicates difficulties.

* Age, sex, indication and LVEF were mutually adjusted and adjusted for SF12GH while SF12 GH, SF12 MCS and SF12 PCS were adjusted for age, sex, indication and LVEF.

Persons receiving social assistance range from those unemployed without labour-market insurance and no other problems than unemployment to people with severe social and psychological problems. Hence, those who were registered at job centres were allocated in the group with labour market–related benefit, while the others were allocated in the group with health-related benefits.

We calculated the proportions of each income category at multiple time points before and after the PCI. RTW was analysed by logistic regression and in time-to-event analyses. Analyses were performed in the whole population as well as in patients who were in the workforce before the PCI. DREAM categories were dichotomised so that the categories “Self-supporting” and “Labour market–related benefit” defined RTW. In Denmark citizen can only receive labour market-related benefit if they can document they are capable and willing to work. We defined RTW as at least four consecutive weeks of no health-related benefits. We defined the week of the PCI as “sick listed” for all patients regardless of their status in DREAM.

**Table 4:** Predictors of RTW measured with c-statistics (area under ROC curve) with 95% CI at 12 weeks and one year after PCI.

	Age:	Sex:	Indication	LVEF	SF12GH:	SF12 MCS	SF12 PCS	All predictors
12 weeks	0.54[0.51;0.58]	0.56[0.53;0.59]	0.57[0.54;0.60]	0.61[0.56;0.64]	0.68[0.64;0.71]	0.66[0.62;0.70]	0.72[0.68;0.76]	0.77[0.74;0.81]
One year	0.58[0.54;0.61]	0.56[0.53;0.59]	0.52[0.49;0.55]	0.59[0.55;0.63]	0.69[0.65;0.73]	0.63[0,58;0.67]	0.71[0.67;0.75]	0.76[0.72;0.80]

For both the time-to-event analysis of RTW and the logistic regression, patients who had permanently left the workforce in the week before the PCI were excluded (n = 591).

### Statistical Methods

Time-to-event data were summarised with a Kaplan Meier plot. The proportional hazard assumption for Cox regression was not met as the hazard ratio changed over time for the variables gender and indication for PCI. Instead, the pseudovalue regression approach was used to examine the cumulative risk ratio at two time points (12 weeks and one year after PCI) [19]. In the pseudovalue approach a new set of observations (the pseudovalues) are generated and used in a generalized linear model. The pseudovalues can take competing events under account. C -statistics (area under ROC curve) for selected predictors were estimated after logistic regression at the two time points [20]. Model fit of the logistic regressions was assessed by Hosmer-Lemeshow goodness-of-fit tests. Estimates were reported with 95% confidence intervals. Data were analysed in STATA, IC version 11 (Stata Corporation, College Station, Texas).

## Results

### Baseline Characteristics

Almost 80% of the patients were men and 32% were treated acute (Table 1). The baseline characteristics were much the same for men and women, except that women were less often overweight or obese and that more women had left the workforce. Patients treated acute were younger, with a higher LVEF, more often smokers and were more often in the workforce (Table 1). The questionnaire was returned by 1323 patients (83.5%). Non- respondents were slightly younger, with lower LVEF and more often not working before the PCI (data not shown).

### Self-reported Health

Four weeks after PCI women rated their health worse than male patients, both in the general health score and in the two component scores (Table 2). Self-rated health was similar in the two indication groups. Compared to the general Danish population in the age of 55–64 years (men and women, respectively), women in the study population rated their mental health one standard deviation lower, while they rated their physical health only a third standard deviation lower [21]. Men rated their mental health half a standard deviation lower, and their physical health nearly as high as the general male population [21].

### Income Status before PCI and during Follow-up

In Figure 1, income sources before and after PCI is summarized. Roughly half of the patients were self-supporting prior to PCI. Four weeks after the PCI, 23% were self-supporting, and this figure increased to 34% after 3 months and to 40% at 1 year. Already 6 months before the PCI, 9% received health-related benefits, and this increased to 14% 4 weeks before the PCI. Four weeks after the PCI, 39% received health-related benefits, which after 12 weeks had decreased to 26% and at 1 year only 13% received health-related benefits. The proportion of early retirement was roughly constant at all measurement times, normal retirement increased due to increasing age.

Restricting the population to those working the week before the PCI (n = 593), 68% were back to work 12 weeks after PCI and 77% after one year (data not shown). Among those receiving health-related benefits at the week before the PCI (n = 362), 36% and 47% returned to work after 12 weeks and one year, respectively (data not shown).

A total of 994 patients were working or only temporarily out of work in the week before the PCI.

Time to return to work for this group stratified on indication is described in the Kaplan-Meier plot in Figure 2.

### Risk Factors of Return to Work

The patients’ report of their general health 4 weeks after the procedure was strongly associated with RTW at both 12 weeks and after one year with a very strong dose-response effect (Table 3). Mental health was nearly as important as the physical health. Low LVEF was also a risk factor at both short and long term. LVEF did not modify the effect of general health, as the estimates were stable within the strata of LVEF (data not shown).

In unadjusted analyses, young age was associated with low RTW shortly after the intervention, but this was not significant in adjusted analyses and the effect diminished after one year (Table 3). Female gender was associated with low RTW especially shortly after the procedure, but also at long term. Patients treated on acute indication had more difficulties in RTW shortly after the procedure compared to elective treated patients, but this difference diminished after one year. Gender and indication did not modify the effect of the other variables on RTW.

In additional analyses, we considered dead and early retirement (voluntarily or health-related) as competing risk factors to the event of interest (RTW) while keeping normal retirement and emigration as censoring variables. This did not change the estimates.

### Predictors of Return to Work

The results of c-statistics (area under ROC curve) after logistic regression are shown in Table 4. SF12 scores, including the single-item SF12 GH, were the best predictors of both 12-week and one-year RTW (0.63–0.72) followed by LVEF (0.59–0.61). As in the time to event analysis of risk factors above, mental health predicts RTW nearly as well as the physical health. Age, gender, and indication (acute/elective) had little importance. Using all predictors in the model revealed c-statistics of 0.77 and 0.76, respectively.

## Discussion

In a population based study of 1585 patient treated with PCI we found that the patients’ self-rated health one month after the procedure was a significant better predictor of RTW compared to other variables including LVEF, both at short (12 weeks) and long (one year) term. Mental health was nearly as important as physical health. The results were robust to stratifications and restrictions.

This study was based on a large cohort of PCI patients with complete register based follow-up of working status. The clinical data from WDHR are complete with respect to individuals [14], and only few values are missing. Self-reported health integrates all the patient’s perceptions and beliefs and because the questionnaire was completed 4 weeks after the PCI, the patients already have some knowledge about the effect of the procedure and the doctor’s advice of sick leave. This could be a part of the causal pathway. The wording in SF-12 is retrospective “In the preceding 4 weeks…”, but for 345 patients the outcome of interest (RTW) occurred before answering the questionnaire. We made an additional analysis excluding these patients, but this changed the c-statistics only marginally.

Missing answers of single items of the SF12 scale resulted in 156 missing values of the mental component score (MCS) and the physical component score (PCS). Analyses based on multiple imputations using available SF-12 items, age, gender and working status did not change estimates or size of confidence intervals in analyses using the two component scores, and consequently the original data were used. A few missing values were present in the variables BMI, LVEF, smoking status and information about diabetes (Table 1). Since these variables originates from a clinical database where clinicians could forget to fill in some items, we consider these variables as missing completely at random (MCAR), since whether or not a variable was missing was not related to the outcome of interest and thus not likely to cause any bias. We had complete follow-up of weekly working status due to full coverage of registers of transfer income in the DREAM database, although there may be misclassifications as sick leave less than 2 weeks does not qualify for national benefit. The grouping of transfer-payment groups may cause misclassification, if a person on labour market–related benefit is not ready to work due to health problems, but fails to report this. However, the group was small and as citizens on labour market–related benefit are strongly encouraged to confirm their readiness to work every week, we consider this a minor problem. Misclassification of the outcome may occur in persons who are not working, but provided income by their spouse or live as rentiers. In Denmark this is rather uncommon as only 2% of the population between 40 and 67 years are without personal income, so we consider it a minor problem.

Measuring RTW as a time-to-event measure has the disadvantage that it ignores relapses if a new sick listing occurs later. A major strength of using the DREAM register in relation to RTW is the ability to establish a measure that captures the RTW dynamically, and not only time to first RTW. In this study 245 persons experienced one or more new sick listings. We made additional analysis by excluding these 245 patients with a dubious outcome, and this enhanced the associations found.

Non-responders had lower LVEF and were less likely to have worked just before the PCI. We repeated the analysis after placing non-responders in the lower groups of the component scores, and this did not change the estimates found for self-reported health.

The proportion of patients working before the PCI was similar to previous findings [22], [23]. RTW among patients working before the PCI compares well with previous studies [4], [22]–[30]. Age [4], [27], [31] and gender [27] have previously been found to predict RTW in mixed populations of heart patients. Another study found neither gender nor age related to RTW [25]. However, Nielsen et al. found that gender modified the effect of low LVEF on RTW [3]. Studies of mortality in heart disease have found that gender differences diminish after adjustment for age [32], but we did not reproduce this with the outcome RTW. Gender differences in sickness absence are well known in Western countries [33], and are suggested to come from both direct and indirect gender effects, such as differences in daily life and social position [34].

Both myocardial infarction prior to the PCI [30] and LVEF [3] has previously been identified as predictors. We found that both acute myocardial infarction and LVEF were predictors; however, the self-reported measures of health were even stronger predictors of RTW.

Self-reported health has previously been found to predict readmissions and mortality after cardiovascular disease [8], [10], [11]. A large Dutch study of 1-year mortality after angiographic procedures found that problems with self-care and low self-rated health were the most powerful predictors among 22 clinical variables and reported that addition of self-reported health improved the model c-statistics from 0.78 to 0.81 [12]. In our study, an addition of the single-item general health from SF12 to the clinical information improves the model c-statistics from 0.66 to 0.73 for RTW at 12 weeks and from 0.66 to 0.75 at 1 year. Adding the complete SF12 score improved the c-statistics even more.

In both the analysis of risk factors and predictors we found that the mental health component score were nearly as important as the physical component scores. A recent review indicated that not only poor physical health, but also poor mental health was associated with adverse prognosis (mortality and rehospitalisation) in hearth disease [35]. Anxiety and depression has previously been found associated with RTW after heart disease [27], [36] and recent work has suggested a close relationship between negative emotions/distressed personality and the risk of incident heart disease as well as poor prognosis [37]–[40].

This study covers nearly all incident PCI patients, under the age of 67 years, originating from a well-defined population in Denmark. The findings are supposed to have high external validity in relation to countries with similar rules and regulations regarding health-related benefits.

Patients’ subjective experiences may differ from clinicians’ views and objective measures such as LVEF. Patient-reported measures and objective measures should complement each other, and thus create a better basis for clinical advice to the patient and risk stratification in RTW.
